# The Effect of Peripheral Magnetic Stimulation on Functional Mobility and Morphology in Cerebral Palsy with Spastic Diplegia: A Randomized Controlled Trial

**DOI:** 10.3390/life15030416

**Published:** 2025-03-07

**Authors:** Kultida Klarod, Oranat Sukkho, Sirirat Kiatkulanusorn, Phurichaya Werasirirat, Chananwan Wutthithanaphokhin, Danguole Satkunskienė, Siraya Lueang-On, Pornpimol Muanjai, Nongnuch Luangpon

**Affiliations:** 1 Department of Physical Therapy, Allied Health Sciences Faculty, Burapha University, Saen Suk, Chonburi 20131, Thailand; kultida@go.buu.ac.th (K.K.); oranat.su@go.buu.ac.th (O.S.); siriratk@go.buu.ac.th (S.K.); phurichayaw@go.buu.ac.th (P.W.); pornpimolm@buu.ac.th (P.M.); 2 Physical Therapy Division, Faculty of Medical Science, Nakhonratchasima College, Nakhonratchasima 30000, Thailand; chananwan.w@gmail.com; 3 Department of Health Promotion and Rehabilitation, Lithuanian Sports University, 44221 Kaunas, Lithuania; danguole.satkunskiene@lsu.lt; 4 Special Education Bureau, PhluTaLuang, Chonburi 20180, Thailand; siraya04@gmail.com

**Keywords:** magnetic therapy, muscle thickness, walking, cerebral palsy

## Abstract

Peripheral magnetic stimulation (PMS) is commonly used for neurological conditions, but its effectiveness in enhancing functional mobility and morphology in children with spastic diplegia remains underexplored. This study assessed the impact of PMS with physical therapy (PT) versus PT alone on mobility and morphology in spastic diplegia. Forty-five children with spastic diplegia (mean age 12.7 ± 3.8 years) were randomly assigned to one of three intervention groups: PMS + PT, PT, or control, with fifteen children in each group. The training was conducted thrice weekly for eight weeks, included muscle morphology assessments, the 30 s sit-to-stand test (30sSTS), functional reach test (FRT), 10 m walk test (10MWT), and 6 min walk test (6MWT). The study revealed increased left quadricep and calf muscle thickness following PMS + PT (d = 0.19, 0.39, respectively; all *p* < 0.05). Improvement in 30sSTS was observed after both PMS + PT (d = 0.56) and PT (d = 1.43). FRT demonstrated increases following both PMS + PT and PT interventions (d = 1.52, 0.93, respectively). Furthermore, improvements were observed in 10MWT following PMS + PT and PT interventions (d = 1.20, 0.78), while PT increased the 6MWT (d = 0.82). The control group showed declines in 10MWT and 6MWT. The treatment significantly impacted FRT, 10MWT, and 6MWT in spastic diplegia. While PMS may not enhance physical capacities beyond PT alone, it may improve FRT and 10MWT outcomes.

## 1. Introduction

Cerebral palsy (CP) is a disorder of individuals whose brains have been compromised, often from an early stage of development, leading to a range of motor and postural difficulties that persist into adulthood. The condition arises from cerebral hypoxia within the motor cortex, resulting in the destruction or underdevelopment of the aforementioned brain regions either prenatally, perinatally, or postnatally. These regions function to control and command the posture and movement of the body [1], leading to patients exhibiting exaggerated reflex responses, muscle stiffness, abnormal muscular tightness, and occasional muscle pain. Symptoms of spastic CP may manifest with gait abnormalities, contingent upon the type and extent of muscular involvement [2]. Spastic diplegia, a subtype of spastic CP, stems from damage to or impairment of the white matter of the brain, termed Periventricular leukomalacia (PVL) and attributed to prenatal hypoxic events occurring around 28 weeks of gestation or in young children [3]. This leads to excessive tightness in the hip and leg muscles, causing toe-walking and a characteristic “scissor gait”, culminating in difficulty or inability to walk without assistive devices [4].

Physical therapy (PT) primarily focuses on managing muscle spasticity, which occurs when muscles work harder than usual, leading to muscle contractures and increased sensitivity of the muscle spindles to stimulation. The shortened muscle contraction and heightened sensitivity of the muscle spindles lead to increased muscle tone and activity. Therefore, the fundamental objectives of rehabilitation and physical therapy are to increase muscle flexibility in overworked muscles, reduce muscle contractures, and decrease muscle activity [5]. Proprioceptive Neuromuscular Facilitation (PNF) strengthening exercises are predominantly employed for the lower extremities and are known to significantly enhance muscle strength and balance. For instance, previous studies have confirmed the efficacy of this neurological approach, demonstrating improvements in trunk control, upper extremity functional skills, selective proximal muscle strength, and both distal upper extremity muscle strength and grip strength [6]. In addition to PNF techniques, static stretching has also been shown to acutely increase muscle–tendon unit elongation in individuals with CP [7].

Repetitive peripheral magnetic stimulation (rPMS) emerges as a viable alternative intervention for managing the condition. PMS targets the peripheral nerves or muscles rather than directly stimulating the brain, thus circumventing the potential risk of triggering epileptic seizures. In older adults with severe upper extremity paresis, rPMS applied over six hemiparetic shoulder–arm muscles led to immediate improvements in motor function [8]. Additionally, this treatment intervention yielded enhancements in the Fugl-Meyer Assessment, Barthel Index scores, as well as muscle strength for grip, elbow flexion, and extension [9], while also demonstrating efficacy in enhancing motor recovery post-stroke, particularly in the subacute stage [10]. The research investigating the effects of PMS in children with neurological deficits has been limited in recent studies. Flamand et al. (2012) suggested that a significant recruitment of sensory afferents induced by stimulation may have the potential to impact central nervous system plasticity in individuals with spastic diparesis. Recent research has also indicated that 3 weeks of functional repetitive neuromuscular magnetic stimulation applied to the gluteus (12 sessions with 12,600 stimuli emitted during 20 min per session) is a safe and well-received neuromodulatory approach. This method shows promise in enhancing the quality of life, particularly in terms of activity and participation, among children and adolescents with bilateral spastic cerebral palsy [11]. Active ankle dorsiflexion and calf flexibility were found to improve after four sessions of bilateral rPMS application to the leg and trunk muscles (tibialis anterior, hamstrings, transverse abdominis, and paraspinal multifidus) in an adult patient with cerebral palsy [12].

Given the dearth of research exploring the effects of PMS combined with PT on morphology and functional mobility in children with spastic diplegia CP, this study endeavors to compare alterations in functional mobility and morphology resulting from PMS combined with PT versus PT alone treatment alongside comparisons with a control group. The study hypothesis posited that PMS would yield greater beneficial effects in enhancing the measured variables. The findings of this study hold significant value for clinical applications in the management of CP. This research would provide valuable insights for clinicians and healthcare practitioners. Such insights could inform the development of more effective and targeted interventions aimed at enhancing the quality of life and functional outcomes for individuals living with CP.

## 2. Materials and Methods

### 2.1. Participants

**Figure 1:**
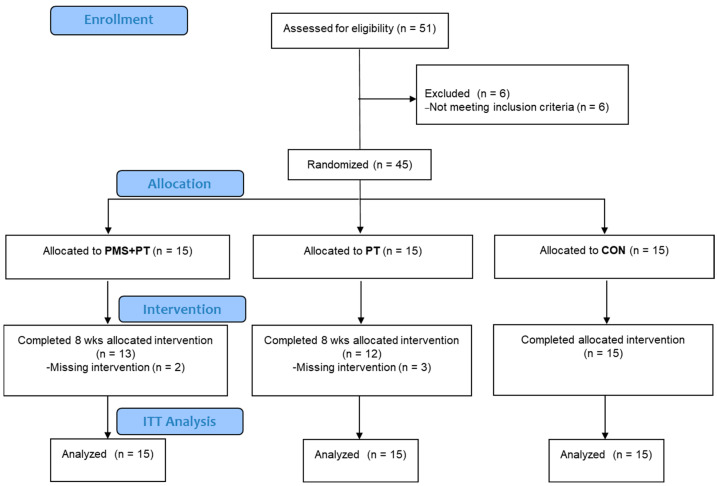
The CONSORT diagram showing the flow of participants in the study.

The study encompassed children diagnosed with CP, specifically presenting symptoms of spastic diplegia, within the age range of 2 to 15 years. Inclusion criteria comprised individuals exhibiting an absence of cranial surgery history, capability to ambulate independently with or without assistive devices (Gross Motor Function Classification System (GMFCS) levels I–III), and the capacity to comprehend and comply with instructions. Exclusion criteria entailed involvement in uncontrolled seizures, presence of metallic implants, active infectious ailments, any congenital disorders such as Down syndrome or fragile-X syndrome, unstable vital signs, or severe cognitive impairment. The cohort comprised 45 participants, divided into three groups: a physical therapy-only (PT) group (n = 15), a cohort receiving combined PMS and PT (PMS + PT) (n = 15), and a control (CON) group consisting of 15 individuals, selected using an envelope selection randomized method, as shown in Figure 1. Ethical approval for the study was granted by the Burapha University Institutional Review Board (IRB1-002/2567) for the Protection of Human Subjects in Research and the Thai Clinical Trials Registry (TCTR20240710002). The study was conducted in compliance with the guidelines of the institutional committee on human research and adhered to the ethical standards outlined in the Helsinki Declaration, as revised in 2013. Informed consent was obtained from the legal guardians of the participants.

### 2.2. Study Design

This study was initially designed to utilize Transcranial Magnetic Stimulation (TMS) as the treatment method. However, due to the presence of seizure conditions in the children during screening via the electroencephalogram by the trained specialist [13], which could lead to adverse outcomes, we decided to discontinue the use of TMS. Instead, we opted to implement PMS as an alternative approach. The study was structured as a randomized controlled trial employing a double-blind design. Measurements were conducted both before and after an 8-week training period, encompassing assessments of muscle ultrasound imaging, the 30 s sit-to-stand test, balance abilities, the 10 m walk test (10MWT), and the 6 min walk test (6MWT). A standardized protocol was followed, with a consistent 5 min rest period between measurements, administered by the same investigators at ChaleoPavana Memorial School (Chonburi Special Education). The study protocol was standardized following the CONSORT checklist and TIDier checklist.

### 2.3. Interventions

#### 2.3.1. Physical Therapy Intervention

The program entailed a PT regimen comprising preparatory exercises aimed at priming the musculoskeletal system. This encompassed warm-up stretches, including trunk rotation and knee-to-chest maneuvers. Subsequently, a series of prolonged passive stretches targeting muscle groups prone to spasticity were executed, with each stretch held for 15–30 s and repeated thrice for the gastrocnemius, soleus, hip adductors, hip flexors, knee flexors, hip extensors, knee extensors, and ankle dorsiflexors. Concurrently, muscle strengthening exercises for the hip extensors, knee extensors, and ankle dorsiflexors were conducted employing tapping/quick stretch maneuvers with manual resistance tailored to individual capacity, comprising 10 repetitions per set, administered across 2 sets. Additionally, PNF lower extremity exercises were incorporated, employing the D1 flexion (D1F) to extension (D1E) and D2 flexion (D2F) to extension (D2E) patterns, with each set comprising 10 repetitions, repeated twice. The entirety of this regimen spanned 30 min per session, administered thrice weekly over a duration of 8 weeks. Moreover, during the final 2 weeks of the program, participants underwent gait training aimed at cultivating a correct gait pattern. This training involved treadmill-based exercises lasting 5–10 min per session, supervised or supported by a skilled physical therapist to ensure proper execution and technique adherence.

#### 2.3.2. Peripheral Magnetic Stimulation Plus Physical Therapy

The participants initially underwent the PT program detailed above. To enhance gait improvement, rPMS was subsequently administered, focusing primarily on strengthening the quadriceps group, as previously indicated [14,15,16]. A coil probe (Transcranial Magnetic Stimulation: type STM9000, EB neuro S.p.A., Firenze, Italy) was applied over the vastus lateralis/medialis and rectus femoris. The rPMS parameters encompassed a frequency of 30 Hz, with an output intensity set at 80% of maximum capacity delivered for a duration of 2 s, followed by an 8 s pause, iterated consecutively for 10 cycles. This protocol was repeated thrice, with a 1 min intermission between repetitions. Stimulation sessions were conducted in triplicate daily, thus totaling 90 stimulatory events per day, administered once daily, 5 days per week, across a span of 8 weeks. This regimen closely adhered to the methodology outlined by Pan et al. (2022) [17], with minor adjustments as warranted.

#### 2.3.3. Control

Participants in the control group received their PT routine once a week.

### 2.4. Outcome Measures

#### 2.4.1. Muscle Ultrasound Imaging

B-mode ultrasonography was conducted using the TE9 series equipment from Shenzhen Mindray Bio-Medical, China. A linear 3.82 cm, 12 MHz probe (neuromuscular preset) was consistently utilized across all participants, with settings configured to 10 MHz frequency, 54 dB gain, and a dynamic range of 60, positioned at a depth of 5 cm. Participants assumed a prone position with the examined foot relaxed for the capture of images of the medial gastrocnemius (MG) muscle, taken 8 cm below the popliteal line. Images of the vastus lateralis (VL) muscle were obtained with participants in a supine position, positioned 10 cm above the superior pole of the patella. During resting periods, two images of each muscle were acquired in both transverse and longitudinal orientations, with the application of gel and minimal pressure on the probe.

Muscle thickness (MT) was analyzed offline utilizing Tracker version 6.0.10 software for the longitudinal images. In this context, MT represented the distance between superficial and deep aponeurosis. An extrapolated line was drawn along visible fascicles between the superficial and deep aponeurosis to estimate fascicle length (FL) [18,19]. Subcutaneous adipose tissue thickness was delineated as the line between the skin and muscle interface. Grayscale analysis was employed to evaluate the raw echo intensity (EI), indicative of muscle quality, in the transverse propagational images, utilizing ImageJ software. The corrected EI was computed as the sum of the raw EI and the subcutaneous adipose thickness multiplied by 40.5278, as per the methodology outlined by Young et al. (2015) [20]. The averages of two images obtained during resting periods for MT, EI, and FL were utilized for subsequent analysis.

#### 2.4.2. The 30 s Sit-to-Stand Test

The 30 s sit-to-stand test assesses functional lower limb muscle strength and performance by quantifying the number of repetitions of sitting and standing within a 30 s timeframe. Each participant sits on a chair without armrests, with feet flat on the ground and hips and knees positioned at 90° and 105°, respectively. Test administrators instruct participants to perform repeated sit-to-stand movements as many times as possible within the 30 s interval without using their arms for support. In cases where participants utilize walking aids, successful completion is determined by the hips extending beyond 75° during the final transition from sitting to standing [21].

#### 2.4.3. The Functional Reach Test

The functional reach test (FRT) is a clinical assessment used to measure postural control by determining the distance between the maximal forward reach of the arm and the starting position while standing. Participants received instructions to stand close to a wall (barefoot) but without touching it. The dominant arm was positioned at the center mark of a measuring stick placed against the wall at the height of the participant’s acromion bone. With the shoulder flexed at a 90-degree angle and the arm extended forward, the evaluator recorded the starting position at the third metacarpophalangeal joint of the hand based on standardized criteria. Participants were then instructed to “reach as far forward as possible without taking a step”. The position of the third metacarpophalangeal joint of the hand at the furthest point reached was recorded. The difference between the starting and ending positions represents the reach distance. The test was repeated three times with a 5 s rest interval between each trial. Results were recorded and averaged for data analysis.

#### 2.4.4. The Timed up and Go Test

The timed up and go test (TUGT) is employed to assess functional dynamic balance. Participants sat on a chair with adjustable height, positioned such that the knees and hips were flexed at 90°, and their feet were placed flat on the floor. Individuals classified at Gross Motor Function Classification System (GMFCS) level III were permitted to use walking aids during the test. During the assessment, participants rose from the chair, walked a distance of 3 m, pivoted around a marked point, returned to the chair, and sat down again [22]. Timing began with the command “go” and concluded when the participant sat back down on the chair. Each participant underwent the test three times, and the best time achieved was recorded in seconds.

#### 2.4.5. The 10 m Walk Test

The 10MWT is conducted to assess the speed of walking. The test was conducted on a 14 m pathway, including the starting and ending points, with a 2 m walk-in section [23]. Before the test, the evaluator instructed, “Please walk to the end at your normal pace”. When the participant was ready, the evaluator commanded, “Ready, go”, and they walked until the end of the pathway. The evaluator started timing when the participant’s first foot crossed the 2 m mark and stopped timing when the first foot crossed the 12 m mark. During the test, the evaluator walked alongside the participant and encouraged them to continue walking. The time taken to reach the midpoint of the 10 m walk was recorded for calculating walking speed.

#### 2.4.6. The 6-Minute Walk Test

The 6MWT is used to measure functional cardiorespiratory capacity. The test was conducted in a 40-square-meter area within a building, with a 1-meter-wide pathway [24]. Cones and ropes were placed to demarcate the four corners both inside and outside the area, with adhesive tape marking a 2 m distance along the pathway. Participants were instructed to walk as far as possible within 6 min, with the option to slow down or stop if necessary. When participants needed to rest, the duration of the rest period was recorded. Participants were encouraged to resume the test if they stopped walking continuously. The distance walked was recorded after the test.

### 2.5. Statistical Analysis

Given the limited number of studies on PMS in CP, the sample size calculation was based on previous research conducted by Gupta et al. (2018), which demonstrated changes in Gross Motor Function Measure (GMFM) scores with an effect size of 0.646 following rTMS. A significance level of 0.05, statistical power of 0.95, and a minimal sample size of 12 were specified using G*Power software (version 3.1.9) [25]. To account for potential dropout events, the sample size was increased by 30% (n = 15) in each group.

All descriptive variables were computed and reported as the mean and standard deviation (SD) or the standard error of the mean (SEM). Normality of the data was initially assessed using the Shapiro–Wilk test. Anthropometrics measurements, GMFM, and TUG across groups were compared using one-way analysis of variance. An intention-to-treat analysis was conducted for this study, and missing data were addressed using the Expectation-Maximization algorithm as recommended by prior research [26]. Time, group, and interaction effects were assessed using mixed ANOVA with Tukey correction applied for post hoc multiple comparisons in cases of significant interactions. The effect size (ES) was quantified using partial eta-squared for repeated measures, with magnitudes defined as small (0.02), moderate (0.13), and large (0.26) [27] and as a Cohen d where the pairwise comparison was made (small (d = 0.2), moderate (d = 0.5), and large (d = 0.8)) [28]. The level of 0.05 was used to determine significance. All statistical analyses were performed using IBM SPSS Statistics for Windows (version 24.0; IBM Corp., Armonk, NY, USA).

## 3. Results

The adherence to protocol in this investigation is visually represented in the CONSORT diagram depicted in Figure 1. Prior to the intervention, the indicators of all three groups were not significantly different, except for the TUG test, where the control group had notably better results (Table 1).

The results of muscle morphology assessment conducted via ultrasound imaging are presented in Table 2. Across all variables, no statistically significant effect of interaction between time and groups were observed. A significant increase in the thickness of the left VL muscle was observed after an 8-week intervention period (F1, 33 = 11.145, *p* = 0.002, η_p_^2^ = 0.21). Both the PT and PMS + PT treatments led to a significant increase in muscle thickness of the left VL (d = 0.99, 0.19, respectively; all *p* < 0.05). Additionally, pairwise comparisons indicated a significant increase in the thickness of the left MG (*p* = 0.024, d = 0.39) and a noticeable improvement in the right MG (*p* = 0.055) in the PMS + PT group. Conversely, measurements pertaining to the muscle quality and FL remained unchanged subsequent to the 8-week intervention period across all groups.

**Figure 2:**
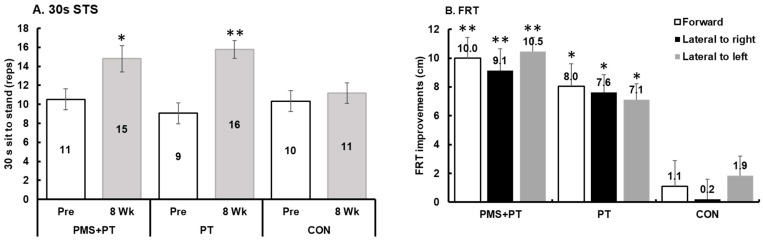
Alteration in functional strength and balance abilities following 8 weeks of interventions: (**A**) 30 s sit-to-stand test; (**B**) functional reach test (FRT). Data are mean and SEM (N = 15). Significant difference compared with pre-measurement (** *p* < 0.001, * *p* < 0.01).

In the assessment of muscle strength using the 30 s sit-to-stand test, there was a significant difference between the groups over time (F2,42 = 3.52, *p* = 0.039, η_p_^2^ = 0.143). Both the PMS + PT (*p* = 0.008, d = 0.56) and PT (*p* < 0.001, d = 1.43) interventions showed a substantial improvement in the 30 s sit-to-stand test. However, following the treatment, only the PT group exhibited significantly higher muscle strength (*p* = 0.048) compared with the control group (Figure 2A). Balance ability was assessed using the TUG and FRT. There was a significant interaction between time and groups in FRT measurements for all tested directions: forward (F2,42 = 7.69, *p* = 0.001, η_p_^2^ = 0.27), lateral to the right (F2,42 = 12.69, *p* < 0.001, η_p_^2^ = 0.38), and lateral to the left (F2,42 = 13.77, *p* < 0.001, η_p_^2^ = 0.40). After treatment, the PMS + PT group showed a significantly higher FRT score than the control group for all tested directions: forward (*p* = 0.007), lateral to the right (*p* = 0.01), and lateral to the left (*p* = 0.002) (Figure 2B). Moreover, there was a significant time effect on FRT measurements for all tested directions: forward (F1,42 = 43.22, *p* < 0.001, η_p_^2^ = 0.51), lateral to the right (F1,42 = 53.15, *p* < 0.001, η_p_^2^ = 0.56), and lateral to the left (F1,42 = 91.90, *p* < 0.001, η_p_^2^ = 0.69). Particularly notable was the considerable clinical improvement in FRT observed following the PMS + PT intervention (d = 1.52, 1.56, 1.76 for forward, lateral to the right, and lateral to the left, respectively, all *p* < 0.001). Additionally, the PT intervention resulted in an average increase of 5.9 cm in FRT scores across all tested directions (d = 0.93, 1.07, 1.11 for forward, lateral to the right, and lateral to the left, respectively, all *p* < 0.01). However, the analysis showed that there were no significant changes in TUG after 8 weeks across all conditions when tested using the Wilcoxon Signed Ranks Test (*p* > 0.05).

**Figure 3:**
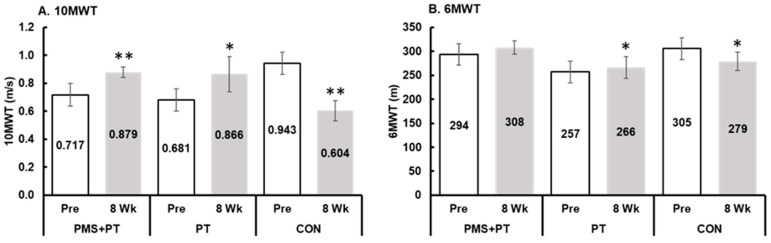
Changes in walking speed and submaximal aerobic capacity following 8 weeks of interventions: (**A**) 10 m walk test (10MWT); (**B**) 6 min walk test (6MWT). Blank bar, pre-test; gray bar, after 8 weeks. Data are mean and SEM (N = 15). Significant difference compared with pre-measurement (** *p* < 0.001, * *p* < 0.01).

The analysis showed a significant interaction between time and groups on the 10 m walking speed (F2,42 = 60.1, *p* < 0.001, η_p_^2^ = 0.74). After the treatment, the PMS + PT group demonstrated a significantly higher walking speed (*p* < 0.05) compared with the control group. Further comparisons showed that both the PMS + PT (*p* < 0.001, d = 1.20) and PT (*p* = 0.006, d = 0.78) interventions led to a significant improvement in walking speed, whereas the control group experienced a decrease in walking speed (*p* < 0.001, d = 2.25) after the 8-week follow-up period, as depicted in Figure 3A. Similar trends were observed for the assessment of submaximal exercise capacity using the 6MWT, with a significant interaction effect (F2,42 = 8.9, *p* = 0.001, η_p_^2^ = 0.30). Specifically, the PT intervention led to significantly enhanced submaximal exercise capacity (*p* = 0.003, d = 0.82), while the control group showed a decrease in submaximal exercise capacity (*p* = 0.004, d = 0.78) after the 8-week follow-up period (Figure 3B).

## 4. Discussion

This study represents the inaugural investigation into the application of rPMS in children diagnosed with spastic diplegia, with a singular focus on the quadriceps musculature. The present study investigated whether the addition of rPMS would enhance morphology and functional capacity in children with spastic diplegia. The primary findings revealed that both PMS + PT and/or PT alone resulted in significant improvements across most parameters, with the exception of the TUG. However, considering the effect size, the results suggest that the inclusion of PMS may trend toward greater improvement, particularly in FRT measurements.

A substantial increase in MT was observed exclusively in the left limb of the PMS + PT group for both the VL and MG muscles, and in the VL muscle for the PT group. Additionally, there was a trend toward an increase in the right MG MT for both groups, although this result approached statistical significance (*p* = 0.055). This finding contrasts with earlier research in which rPMS was applied to four upper extremity muscle groups at intensities of 35% and 45% with a frequency of 30 Hz over a three-week period [10]. That study reported only a mild, statistically non-significant increase in the cross-sectional area (CSA) of the extensor digitorum in individuals with post-stroke hemiparesis. Furthermore, a previous study found no change in rectus femoris CSA following a brief two-week period of rPMS in acute stroke patients [29]. Our observation suggests a potential role for rPMS in the prevention or mitigation of muscle atrophy, paralleling similar effects observed with neuromuscular electrical stimulation (NMES) in preventing loss of muscle mass [30]. In agreement with the previous investigation has reported significant increases in the MT of VL following rPMS application at maximum device intensity in healthy sub-jects, suggesting that rPMS induces muscle expansion via repetitive muscle contractions [31]. However, the observed morphological changes resulting from the PT program may necessitate the consideration of additional factors, suggesting that a progressive increase in load or the duration of intervention may be required to discern any morphological changes in the musculature, as indicated by previous investigations spanning durations of 5 to 12 weeks [32,33].

The PT program implemented in this study effectively enhanced functional leg strength, as evidenced by improvements in the 30sSTS. This success can be attributed to the comprehensive nature of the PT regimen, which targeted various muscle groups with the intent of strengthening, consistent with findings from previous studies investigating neuro-physical therapy techniques [6,33]. The enhanced performance observed following PNF exercises may also be ascribed to the incorporation of principles such as irradiation, spatiotemporal summation, and stretch reflex modulation [34]. Moreover, the supplementary strength improvement induced by rPMS was also evident in this study. Prior research has suggested that rPMS may serve as an adjunctive intervention, potentially providing super-additive effects to enhance the outcomes of conventional treatments in neurological conditions [35]. Several mechanisms have been proposed to elucidate the strength-enhancing effects of rPMS, including the facilitation of motor cortex (M1) plasticity and motricity stimulation [12], reduction in GABAergic inhibition, induction of brain plasticity within the sensorimotor cortex [36], direct activation of sensorimotor nerve fibers, and indirect stimulation of mechanoreceptors during rhythmic contraction and relaxation [37]. Moreover, rPMS has been shown to increase excitability within the cortico-motor pathway, leading to significant activations within the sensorimotor network, cerebellum, and thalamus [38]. The resultant strengthening effect from rPMS has been demonstrated to contribute to functional improvements in patients with spastic paralysis. Nevertheless, due to the lack of standardized recommendations for the utilization of rPMS in children with spastic diplegia, the superior effects of PMS + PT were not realized in the present study.

The functional balance, as measured by the FRT, demonstrated substantial improvement following the conventional PT program in this study. This enhancement can be attributed to the efficacy of PNF exercises, which have been proposed as an alternative to task-oriented training. PNF has been shown to enhance trunk control [6], increase muscle strength [34], and improve the coordination of the lower limbs [39]. Moreover, in a systematic review evaluating clinical tools for assessing balance in children with CP, a variant of the FRT forward task demonstrated moderate evidence for reliability [40]. Children with spastic CP often exhibit musculoskeletal impairments that impede the backward translation of the knee and pelvis, including calf spasticity and/or contracture, deficient eccentric muscle control, and inadequate coordination of selective motor control between agonist and antagonist muscles for the knee and ankle [41]. The adjunct PT program employed in this study effectively mitigated these impairments, resulting in improvements in the FRT scores. Furthermore, the findings of this study suggest the potential recommendation for incorporating rPMS alongside PT programs for managing balance in CP. This suggestion is supported by the higher effect size observed in FRT measurements with the addition of rPMS, indicating its potential to enhance muscle strength or function and subsequently influence balance abilities. Previous research lends further support to this recommendation, as evidenced by the significant increase in tandem standing time observed in the rPMS group following hip replacement surgery [42]. However, it is noteworthy that the treatment in this study did not yield significant effects on the TUG test, potentially due to the multifaceted nature of this assessment, which encompasses balance, anticipatory postural control, and functional mobility [43]. Future studies may consider modifying the PNF protocol to specifically target trunk or pelvic control, as this may have a pronounced impact on improving TUG performance.

An additional effect that may contribute to the improvement of stimulated muscle strength and function is the reduction in spasticity induced by rPMS. Studies have demonstrated a substantial reduction in tendon reflex and compound muscle action potential amplitude following 5 Hz rPMS treatment, suggesting that effective therapy may incorporate rPMS to alleviate muscular stiffness and enhance mobility, thereby potentially improving gait and posture control in individuals afflicted with spasticity [44]. Further-more, research supports the notion that rPMS leads to a reduction in tendon reflex response, particularly in the soleus muscle, when stimulated at the posterior tibial nerve. This reduction is speculated to result from modulation within the spinal and/or supraspinal circuits, leading to a decrease in spinal reflex excitability [45]. The spasticity reduction induced by rPMS offers additional benefits in enhancing motor function and activities of daily living in individuals with spastic paralysis, as indicated by findings from meta-analyses [17]. Therefore, the spasticity-reducing effects of rPMS may complement its role in improving muscle strength and function, ultimately contributing to enhanced motor performance and functional outcomes in individuals with neurological conditions characterized by spasticity.

Functional decline in terms of walking speed and endurance was clinically observed in the control group after repeated measurements at 8 weeks in the present study. Such decline could potentially impact their independent living ability and overall quality of life. Conversely, these measurements significantly improved following the PT program. The utilization of PNF exercises in the PT regimen is noteworthy, as PNF patterns typically involve spiral and diagonal movements, thereby emphasizing functional training aimed at enhancing trunk stability and balance in lateral directions [46]. PNF has been demonstrated as an effective intervention for decreasing muscle spasticity, improving lower-limb function, and enhancing body balance, which may ultimately contribute to increased gait speed [47]. The improvements observed in the 6MWT response to the PT program align with findings from previous research. For instance, a 16-week combined stretching and strength training program in children with CP at GMFCS levels I and II resulted in an average increase of 45.7 m in the 6MWT [48], albeit a lesser increase was noted in our study (27.3 m (95%CI: 8.7, 45.8 m)). Enhanced walking ability has been associated with improved fitness levels, subsequently reducing the sense of fatigue [49]. Notably, isometric plantar flexor strength has been identified as a critical independent variable related to the distance walked in the 6MWT [50]. However, individual responses varied, and this change was not consistently observed in the group receiving PMS + PT treatment. While there was an improvement in walking speed, the beneficial effect of rPMS on walking endurance was not supported by the results of this study. Conversely, top-down stimulation treatment, such as rTMS, has shown promise in improving walking ability. Studies have reported improvements in the 10MWT and 6MWT as well as reductions in muscle tone following rTMS intervention in children with hemiplegic CP [51]. These findings suggest enhancements in ankle joint control, lower limb weight-bearing ability, coordination, and stability, which can collectively improve walking efficiency and endurance [52].

The validity of this study is supported by its design as a randomized controlled trial (RCT). Both PMS + PT and PT alone led to clinically significant improvements in muscle thickness, balance, and leg functional ability. However, based on the effect size, the results suggest that the inclusion of PMS may yield greater improvements, particularly in FRT measurements. These findings indicate that PMS could be considered as an adjunctive therapy for children who are susceptible to balance impairments, which, in turn, affect their overall functional ability.

### Limitations

The present study is subject to several limitations that warrant consideration. Firstly, the utilization of static rPMS could be reconsidered in favor of a protocol involving magnetic stimulation applied concurrently during physical exercises, as demonstrated by Novak et al. (2020) [52]. Such an approach has been associated with high satisfaction levels and no reported adverse effects [14]. Secondly, the study’s duration of treatment and follow-up may be insufficient to fully ascertain the long-term effects of rPMS in children with CP. Extending the treatment and follow-up period could provide valuable insights into the enduring benefits of rPMS, particularly considering the heightened brain plasticity observed during the subacute stage of CP onset, with treatment responses diminishing in the chronic and sequelae stages [53]. Lastly, exploring the application of rPMS to other muscles, such as the gluteus and calf, may yield additional functional improvements. By targeting a broader range of musculature, rPMS has the potential to induce more comprehensive enhancements in functional movement.

## 5. Conclusions

In summary, leg muscle thickness and the 30sSTS, FRT, and 10MWT demonstrated clinical improvements following an 8-week PMS + PT and PT program. However, the application of rPMS may not confer additional benefits in enhancing physical capacities in spastic diplegia when compared with PT-alone interventions. Despite this, considering the effect size, the addition of rPMS may trend toward greater improvement, particularly in FRT and 10MWT measurements. The effectiveness of rPMS in other muscle groups as well as the potential need for adjustments to the treatment protocol warrant further investigation in patients with cerebral palsy.

## Figures and Tables

**Table 1:** Characteristics of participants.

	PMS + PT (N = 15)	PT (N = 15)	CON (N = 15)	*p*-Value
Gender (B/G)	9/6	7/8	11/4	0.388
Age (years)	13.6 ± 2.8	11.9 ± 3.1	12.6 ± 2.6	0.280
Weight (kg)	44.4 ±17.1	35.5 ± 12.0	40.2 ± 15.2	0.271
Height (cm)	143.8 ± 17.9	138.0 ± 15.3	145.1 ± 14.0	0.432
BMI (kg/m^2^)	21.7 ± 9.5	18.3 ± 4.2	18.4 ± 4.1	0.268
GMFCS (I/II/III) *	6/2/7	6/5/4	11/1/3	0.160
TUG (s)	14.36 (8)	19.05 (65)	11.1 (7)	0.047 *

Data are mean and SD or median (IQR). BMI, body mass index; GMFCS, Gross Motor Function Classification System. * GMFCS values are calculated using Fisher’s exact probability method. TUG, timed up and go.

**Table 2:** Muscle ultrasound imaging as a result of treatment intervention.

USI	PMS + PT (N = 15)			PT (N = 15)			CON (N = 15)		
	Pre	Post 8 Weeks	*p*	Pre	Post 8 Weeks	*p*	Pre	Post 8 Weeks	*p*
VL MT (cm)									
RT.	1.80 ± 0.35	1.83 ± 0.37	0.378	1.68 ± 0.29	1.65 ± 0.29	0.353	1.86 ± 0.39	1.86 ± 0.41	0.961
LT.	1.68 ± 0.33	1.74 ± 0.29	0.049 *	1.69 ± 0.22	1.75 ± 0.25	0.037 *	1.68 ± 0.45	1.72 ± 0.47	0.193
MG MT (cm)									
RT.	1.17 ± 0.29	1.22 ± 0.26	0.055	1.01 ± 0.33	1.06 ± 0.32	0.055	1.34 ± 0.18	1.33 ± 0.21	0.542
LT.	1.16 ± 0.23	1.23 ± 0.25	0.024 *	1.03 ± 0.31	1.05 ± 0.29	0.468	1.29 ± 0.31	1.29 ± 0.32	0.884
VL EI (A.U.)									
RT.	115.9 ± 23.5	121.1 ± 23.6	0.093	117.6 ± 16.98	117.15 ± 17.27	0.896	113.7 ± 25.08	116.1 ± 28.79	0.428
LT.	123.2 ± 30.2	124.6 ± 32.7	0.529	116.3 ±12.9	116.2 ± 15.1	0.955	112.9 ± 15.3	116.5 ± 17.6	0.177
MG EI (A.U.)									
RT.	113.4 ± 15.9	119.1 ± 21.1	0.073	119.0 ± 11.1	118.3 ± 23.3	0.797	115.5 ± 20.4	120.5 ± 29.6	0.115
LT.	110.3 ± 19.5	113.2 ± 15.3	0.369	120.1 ± 14.3	124.7 ± 22.6	0.142	117.5 ± 22.5	118.8 ± 27.4	0.671
VL FL (cm)									
RT.	6.71 ± 1.44	6.72 ± 1.23	0.162	6.44 ± 1.38	6.43 ± 1.20	0.137	7.74 ± 1.28	7.72 ± 1.05	0.369
LT.	6.24 ± 1.43	5.91 ± 1.28	0.831	6.62 ± 1.49	6.75 ± 1.60	0.569	6.66 ± 1.37	6.72 ± 0.99	0.876
MG FL (cm)									
RT.	4.12 ± 1.51	4.32 ± 1.43	0.055	3.93 ± 1.14	4.03 ± 1.16	0.315	4.25 ± 0.95	4.23 ± 0.98	0.873
LT.	3.71 ± 0.84	3.74 ± 1.33	0.787	4.05 ± 1.17	4.07 ± 1.25	0.874	3.55 ± 1.25	3.59 ± 1.25	0.713

Data are mean and SD. VL, vastus lateralis; MT, muscle thickness; MG, medial gastrocnemius; EI, echo intensity; FL, fascicle length. No significant changes were seen in the present study. * Significant difference compared to Pre-intervention (*p* < 0.05).

## Data Availability

The data for this study are available upon request.
